# What Limits Cardiac Performance during Exercise in Normal Subjects and in Healthy Fontan Patients?

**DOI:** 10.1155/2010/791291

**Published:** 2010-09-07

**Authors:** André La Gerche, Marc Gewillig

**Affiliations:** ^1^University Hospital, Catholic University of Leuven, 3000 Leuven, Belgium; ^2^St Vincent's Hospital, University of Melbourne, 3065 Fitzroy, Australia

## Abstract

Exercise is an important determinant of health but is significantly reduced in the patient with a univentricular circulation. Normal exercise physiology mandates an increase in pulmonary artery pressures which places an increased work demand on the right ventricle (RV). In a biventricular circulation with pathological increases in pulmonary vascular resistance and/or reductions in RV function, exercise-induced augmentation of cardiac output is limited. Left ventricular preload reserve is dependent upon flow through the pulmonary circulation and this requires adequate RV performance. In the Fontan patient, the reasons for exercise intolerance are complex. In those patients with myocardial dysfunction or other pathologies of the circulatory components, it is likely that these abnormalities serve as a limitation to cardiac performance during exercise. However, in the healthy Fontan patient, it may be the absence of a sub-pulmonary pump which limits normal increases in pulmonary pressures, trans-pulmonary flow requirements and cardiac output. If so, performance will be exquisitely dependent on pulmonary vascular resistance. This provides a potential explanation as to why pulmonary vasodilators may improve exercise tolerance. As has recently been demonstrated, these agents may offer an important new treatment strategy which directly addresses the physiological limitations in the Fontan patient.

## 1. Introduction

Exercise is an important determinant of health and provides numerous cardiovascular, psychological, and prognostic benefits [1]. The Fontan operation, and its refinements, have proved a major success with a majority of patients with univentricular malformations now surviving into adulthood with a good quality of life [2]. However, exercise tolerance is significantly reduced and the factors responsible for this exertional limitation are incompletely understood. This paper aims to provide a unique perspective on the limitations of the Fontan circulation by focusing on exercise constraints in a normal biventricular circulation. Rather than describing abnormalities within the components of a Fontan circulation, we will focus on highlighting the importance of that which is missing—a prepulmonary pump. Popular models of exercise physiology have concentrated on the systemic circulation and the left ventricle (LV) as the primary determinant of cardiac output (CO) augmentation during physical exertion [3]. These models are likely to be accurate in patients with heart failure. However, in the healthy subject, there is a large reserve for exercise-induced augmentation of the systemic ventricle and vasculature. A less commonly cited determinant of cardiac performance during exercise is the “lesser circulation” of the RV and the pulmonary circulation [4–6]. We will discuss the evidence supporting the concept that the right ventricle (RV) is a determinant of exercise performance and that, in its absence, pulmonary vascular resistance becomes a critical limitation. The recent availability of relatively specific pulmonary vasodilators has given these physiological discussions immediate clinical relevance. The potential benefit of these agents [7] is consistent with physiological theory and represents an exciting avenue of investigation in Fontan patients.

## 2. Cardiac Output Augmentation during Exercise in the Normal Heart

Exercise performance is defined by the ability of the body's working muscles to utilize oxygen, and this can be measured by VO_2_ max on cardiorespiratory testing. It has been demonstrated that the skeletal muscles' ability to utilize oxygen far exceeds the capacity of the cardiovascular system to deliver oxygen [8]. Thus, cardiac output (CO) becomes the limiting step, and explains 70%–85% of the variance in VO_2_ max with the remainder being determined by peripheral factors such as mitochondrial density which enables greater oxygen extraction [9]. In healthy subjects, CO may be expected to increase 3- to 5-fold whilst increases are even greater in athletes. Cardiac output is enhanced by (1) greater preload, (2) increased heart rate, (3) increased myocardial contractility, and (4) reduced afterload during exercise, and both ventricles need to generate the same stroke volume. Thus we can speculate as to what is the “weak link” by asking which factor is closest to its physiological limit during exercise. Conventional teaching is that it is the LV and its interaction with the systemic circulation which determines cardiac output. However, LV functional measures and measures of systemic vascular tone have not been shown to predict exercise performance as might be expected if they were the limitation of exercise performance [10–12]. 

### 2.1. Preload and Exercise

Preload may be defined as the passive stretch applied to the myocardium prior to the initiation of active contraction. According to the Frank-Starling mechanism, increases in preload augment cardiac output. The extent to which this mechanism contributes to exercise-induced CO augmentation remains a point of debate. Studies using radionuclide ventriculography have reported large increases in LV end-diastolic volume during exercise and minimal or no decrease in LV end-systolic volume. Warburton et al. [13, 14] suggested that this increase in LV preload was the dominant means by which CO increased during exercise, especially in well-trained athletes [13, 14]. However, more recent exercise studies using 2D echocardiography [3, 15–19] and cardiac magnetic resonance (CMR) [20–22] have found little or no increase in LV end-diastolic volume and that augmentation of stroke volume is primarily due to decreases in LV end-systolic volume. Assimilating these contrasting findings is difficult, but perhaps a reasonable summary may be that enhanced LV preload contributes to, but is not the sole determinant of, exercise-induced increases in stroke volume.

Accepting the contribution of LV preload in cardiac output augmentation, the question arises as to what determines LV preload? This is seldom discussed in the literature. It is often assumed that the LV generates flow which then serves as its own preload with, perhaps, some facilitation from skeletal muscle acting to enhance venous return. With a few notable exceptions [4–6, 23], the RV and pulmonary circulation have rarely been considered as potential constraints on LV filling in normal exercise physiology. We speculate that its importance may have been underappreciated. At least in settings where RV afterload is increased, reduced flow in the presystemic circulation can result in reduced LV preload and, thus, cardiac output limitation. Holverda et al. [20, 21] demonstrated reduced LV filling during exercise in patients with pulmonary hypertension and obstructive airways disease. Also, the demonstration of improved exercise tolerance with pulmonary vasodilators provides some indirect affirmation of this concept. It has been shown that sildenafil [24, 25] and bosentan [26] can increase exercise performance at altitude or during hypoxic exercise although no benefit has been demonstrated in normoxic settings. It was concluded by Faoro et al. [26] that the pulmonary circulation provides a limitation to cardiac output when there is hypoxic pulmonary vasoconstriction. The lack of efficacy of selective pulmonary vasodilators during normoxic exercise may suggest that pulmonary vasodilation is close to maximal in this setting [27].

Frequently, exercise intolerance is attributed to diastolic dysfunction, and it is implied that the LV filling impairment is due to pathology of the LV myocardium. However, it is important to note that it is extremely difficult to differentiate impaired filling due to myocardial stiffness from that due to reduced preload when using noninvasive measures [28]. Echocardiographic measures are similar when there is a lack of “push” from reduced preload and when there is a lack of “suck” from LV impairment. Puwanant et al. described reductions in traditional and novel diastolic measures in patients with pulmonary hypertension despite relatively normal LV function [28]. Hart et al. reported measures of diastolic dysfunction which were associated with preload reduction following marathon running. These changes normalized immediately with preload augmentation by leg lifting [29]. Finally, in a large cohort of patients with obstructive pulmonary disease, Barr et al. attributed impaired LV filling to preload reduction through increased pulmonary vascular resistance rather than myocardial stiffness resulting from hypoxia or hypertension [30].

### 2.2. Afterload and Exercise

Afterload can be defined as the myocardial stress achieved during contraction, the major determinants being systolic pressure, cavity size, and wall thickness [31]. Decreases in LV afterload serve to increase stroke volume and this has been emphasized as an important source of cardiac output modulation in popular models of exercise physiology. It has even been suggested that the exercise-induced decreases in systemic vascular resistance (due to vasodilation in skeletal muscle) is the primary determinant of CO augmentation, and that the resultant increases in heart rate and contractility are simply a compensation to maintain systemic blood pressures [32, 33]. However, it has been well established that the capacity for peripheral vasodilation far exceeds the capacity for cardiac output augmentation, implying that the limitation to cardiac output is upstream of the peripheral circulation [34]. 

Characterization of RV afterload during exercise is more difficult because its measurement requires invasive pulmonary artery catheterization or echocardiographic estimates, both of which can be challenging during exercise. Whilst it is commonly stated that pulmonary artery pressures increase only slightly with exercise [35, 36], multiple studies have reported considerable increases in pulmonary artery pressures [4, 23, 37–42]. Kovacs et al. [42] combined data from nearly 200 patients in invasive studies to demonstrate that mean pulmonary artery pressures increased in a linear manner with exercise which explains why relatively high pressures have been measured in those with good exercise tolerance. Argiento et al. [37] described exercise-induced increases in systolic pulmonary artery pressures that frequently exceeded 60 mmHg in healthy subjects whilst Bidart et al. [39] reported even greater increases in athletes. It may be that these increases in pulmonary artery pressures are a result of an inability for the pulmonary vasculature to reduce its resistance sufficiently to compensate for the increased CO of exercise. This concept is best summarized by the simplified Poiseuille's relation in which CO is proportional to pressure and inversely to resistance [3]. Pulmonary vascular resistance is very low at rest and previous studies have suggested that its capacity for reduction is only 20%–50% [23, 35, 43]. This represents a marked difference to the systemic circulation where greater reductions in peripheral resistance moderate increases in LV afterload (see Table 1).

### 2.3. Contractility and Exercise

Contractility is an intrinsic property of the myocardium, is load independent, and is most frequently derived from invasive indices such as the slope of the end-systolic pressure volume relation or the preload recruitable stroke work [44]. There are no studies which have assessed and compared LV and RV contractility during exercise in healthy subjects. At rest, RV measures of mass [45] and contractility [46] are one-third to one-fifth those of the LV and this appropriately matches the pressure requirements of each [47]. However, when RV afterload increases, RV contractility may be unable to maintain ventriculo-arterial coupling such that cardiac output declines. MacNee [48] described marked reductions in RV stroke volume (~30%) when pulmonary artery pressures doubled and compared this with only slight decreases in LV stroke volume with physiological increases in mean arterial pressures. During exercise, Morrison et al. [23] used first-pass radionuclide ventriculography and invasive pressure measures to demonstrate a progressive improvement in RV ejection fraction with exercise-induced reductions in pulmonary vascular resistance. However, in comparison with the systemic circulation, increases in pulmonary vascular pressures were relatively greater, and reductions in vascular resistance were less. This would suggest that exercise results in a disproportionate workload for the RV, a concept which is further supported by the demonstration that cardiac fatigue predominantly affects the RV after intense prolonged exercise [49, 50]. Therefore, there are reasonable grounds to contend that exercise-induced increases in RV load represent a potential for cardiac output limitation. For cardiac output to be maintained during exercise, the RV must greatly augment its contractility, possibly more so than the LV. As will be discussed, this is problematic for exercise performance when RV function is reduced or absent.

### 2.4. Heart Rate and Exercise

Heart rate is a major determinant of cardiac output, particularly in the later phases of exercise when stroke volume augmentation plateaus [3]. It is, however, inefficient for the heart rate to increase such that LV filling is impaired. Thus, although the factors which determine maximal heart rate during exercise are incompletely understood, it is likely that cardiopulmonary baroreceptor activation contributes to maintaining an appropriate balance between LV preload and heart rate during exercise [51].

## 3. The Healthy Fontan Patient and Exercise: How Is Reduced Exercise Capacity Explained?

**Figure 1:**
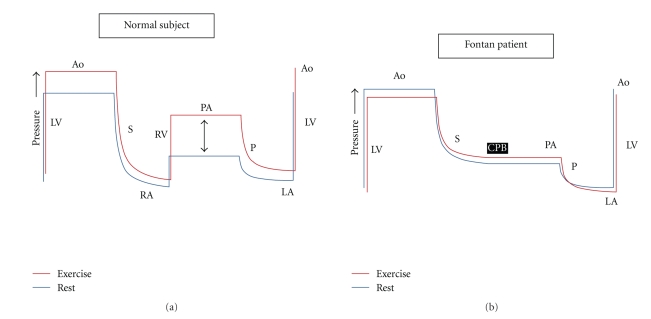
Theoretical schema to illustrate circulatory pressure changes in normal and Fontan patients at rest and during exercise. In the normal circulation, pressure is generated in the systemic ventricle (LV) to produce flow in the aorta (Ao) and systemic circulation (S). Pressure dissipates across the systemic microcirculation such that right atrial (RA) pressures are low. The pre-pulmonary pump (RV) provides the pressure to generate the flow in the pulmonary artery (PA) which then dissipates in the pulmonary circulation (P) but is sufficient to maintain preload in the left atrium (LA). During exercise, systemic vascular resistance falls such that there is little increase in mean LV pressure requirements. However, more substantial pressure increases are required in the RV (purple arrow), and these pressure requirements increase with exercise intensity. In the Fontan patient (below), the cavopulmonary bypass (CPB) does not provide any contractile force and, therefore, flow through the pulmonary circulation is dependent on the pressure difference between the RA and LA. During exercise, trans-pulmonary flow can only be augmented by reductions in pulmonary vascular resistance. Beyond mild to moderate exercise, pulmonary vasodilation is maximal and flow increases require a pre-pulmonary pump. Without this, pulmonary pressure does not rise, trans-pulmonary flow does not increase, LA pressure (preload) does not increase, and cardiac output cannot supply the metabolic demands of exercise.

In the Fontan circulation, the systemic and pulmonary vascular beds are connected in series without the presence of a pre-pulmonary pump to add forward energy to flow through the lungs (Figure 1). Typically CO in a Fontan circulation at rest is decreased to 70% (range 50%–80%) of normal and an increase in peripheral oxygen utilization compensates for this reduction in oxygen supply [52]. During exercise, oxygen utilization remains normal or supra-normal but maximal CO is approximately half that of normal [53]. Control of cardiac output in a Fontan circulation is complex with a different interplay of contractility, afterload, preload, and heart rate [54]. The implications of exercise on each of these variables will be discussed and contrasted with that already described for normal subjects.

### 3.1. Preload and Exercise in the Fontan Patient

There is limited data in which LV filling during exercise has been assessed in the Fontan patient. More frequently, studies have assessed the hemodynamic response to low-dose dobutamine as an exercise surrogate. Senzaki et al. [55, 56] have twice demonstrated limitations in preload reserve in this setting. They demonstrated a significant 25% reduction in LV end-diastolic pressure in the Fontan patient whereas there was no change in subjects with normal circulation [56]. Using variables derived from ventricular pressure-area hybrid loops, they found that cardiac index was dependent upon preload (end-diastolic area) and not contractility (ventricular elastance) or afterload (arterial elastance). Thus, inadequate preload reserve was implicated in the failure for CO to augment with *β*-adrenergic stimulation. Robbers-Visser et al. [57] used CMR imaging to provide a volumetric assessment of dobutamine-induced cardiac augmentation in a young group of patients with Fontan circulation. They described a modest increase in CO which was entirely due to increasing heart rate. Stroke volume was unchanged due to the fact that end-diastolic volumes decreased to the same extent as end-systolic volumes. This contrasts with the normal physiological response to dobutamine in which stroke volume increases due to a reduction in end-systolic volume and *preserved* end-diastolic volumes [58, 59]. Thus, CO limitation in the Fontan patient was again attributed to inadequate preload reserve suggesting that the constraint to exercise is “up-stream” of the LV.

As discussed previously, measures of impaired filling should not be regarded as synonymous with LV pathology. Preload reduction and myocardial relaxation impairment will manifest similarly on non-invasive measures such as echocardiography. This is well illustrated in a recent study performed by Goldstein et al. [60] in which echocardiographic indices of diastolic dysfunction were demonstrable in 16 of 28 young Fontan patients. However, hemodynamic data performed in a subset of these patients during exercise demonstrated normal LV filling pressures which did not increase with exercise, a finding which is inconsistent with the raised filling pressures expected with diastolic dysfunction [61, 62]. Rather, these findings again reinforce the concept of preload inadequacy whereby the normal exercise-induced augmentation in LV filling pressures [42] is absent in the Fontan patient.

### 3.2. Afterload and Exercise in the Fontan Patient

In the Fontan patient during exercise, the reduction in systemic vascular resistance is less than in the normal subject [52]. It is likely that this represents a secondary phenomenon which attempts to maintain blood pressure when CO augmentation is reduced. This is achieved by an increase in sympathetic vasomotor tone [63] but is not always sufficient and BP may still fall in the Fontan patient during exercise [52]. Thus the relatively greater systemic afterload in the Fontan patient is a consequence, rather than cause, of reduced output. 

As described earlier, normal exercise is associated with an increase in stroke volume and pulmonary artery pressures. A pre-pulmonary pump is required to generate the pressure and flow which enables adequate LV filling during exercise. Without a pre-pulmonary pump, and without adequate preload, cardiac output cannot increase during exercise. Figure 1 provides a graphic summary of this proposed physiology.

Indirect evidence for our proposition that the pulmonary circulation serves as a limitation to exercise in the Fontan patient is provided by the demonstration that exercise tolerance is improved with pulmonary vasodilators. Giardini et al. [7] studied the exercise response in 18 Fontan patients before and after a single oral dose of sildenafil as compared with a group of 9 control Fontan patients. An improvement in VO_2_ max (9.4 ± 5.2%) and cardiac index (10.0 ± 7.2%) was demonstrable in those who took sildenafil whilst these measures were unchanged in those who did not receive treatment. Pulmonary vasodilator therapy warrants further investigation in Fontan patients given its potential to address a likely source of limitation to exercise.

### 3.3. Contractility and Exercise in the Fontan Patient

**Figure 2:**
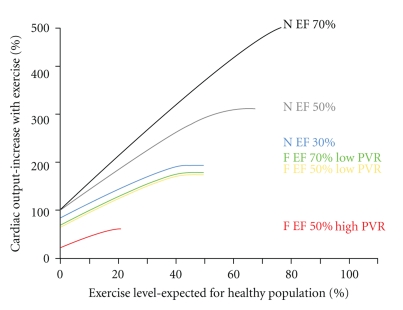
Relationship of output during exercise, pulmonary vascular resistance (PVR), and ventricular function. Cardiac output can increase 5-fold in a normal (N) subject with a biventricular circuit. If ventricular function is impaired, this will first result in decreased maximal output and subsequently in reduced output at low level of exercise. In Fontan patients (F) output is more influenced by PVR than by ventricular function but all have significantly impaired exercise capacity. EF: ejection fraction.

There is limited evidence to suggest that contractility serves as a limitation to CO in the Fontan patient, with the exception of those patients with advanced ventricular dysfunction [54]. As described earlier, Senzaki et al. [55] used hybrid measures of ventricular elastance to demonstrate that contractility and contractile reserve were largely preserved in the Fontan patient. Studies that have suggested reduced systolic performance in the Fontan ventricle have frequently based conclusions on measures which are load dependent [64]. Furthermore, if abnormalities of the systemic ventricle and the peripheral circulation were a cause of exercise limitation, a benefit in exercise tolerance might be expected with ACE inhibitor therapy but this has not been demonstrated [65]. Figure 2 summarizes the relative contribution of LV function and pulmonary vascular resistance to exercise-induced increases in cardiac output.

### 3.4. Heart Rate and Exercise in the Fontan Patient

In the Fontan patient, atrial pacing at increased rates does not result in the augmentation of cardiac output that is seen in normal subjects. Rather, there is a proportional reduction in stroke volume which illustrates heart rate has a less direct effect on CO than in a normal circulation [66]. During exercise, Fontan patients exhibit chronotropic incompetence with a heart rate consistently lower than normal controls, and this has typically been attributed to abnormal reflex control of heart rate or adrenergic dysfunction [67–71]. However, preload inadequacy in the Fontan patient may lead to reducing stroke volume if the ventricular filling time decreases with increasing heart rate. Thus, it is possible that chronotropic incompetence is an *adaptive *response to prevent hemodynamic compromise in patients with limited preload, such as in the Fontan patient [72, 73].

## 4. Summary

Traditional models of exercise physiology have emphasized the performance of the systemic ventricle and systemic circulation. In the Fontan patient with pathology of the systemic ventricle, these abnormalities will largely explain exercise limitation. However, in the healthy Fontan patient, limitation is defined by upstream factors which determine systemic ventricular filling. Inadequate preload reserve has been demonstrated to limit cardiac augmentation caused by ionotropic stimulation and it is likely that this mechanism also explains cardiac limitation during exercise. At rest, pulmonary vascular resistance and pressure are low. During exercise, increases in flow mandate an increase in pressure unless resistance can fall proportionately. Substantial reserve in the systemic circulation moderates pressure increases whilst, in the pulmonary circulation, there is evidence that the capacity for further vasodilation is limited. In a normal biventricular circulation, this results in greater pressure and work demands for the RV during exercise. However, in the Fontan patient, there is no RV to provide this work demand and hence flow, LV filling and CO are unable to increase normally. 

This discussion of Fontan physiology has important clinical implications. Treatment regimens are most likely to be efficacious if they address the part of the circulation which serves as the greatest source of limitation. Classical heart failure regimens have demonstrated poor efficacy in Fontan patients but this may be expected if the systemic circulation is not the primary source of limitation. Selective pulmonary vasodilators target a potential “weak link” in the Fontan circuit. Early results with these agents have offered promise and may prove an important means of improving exercise tolerance.

## Figures and Tables

**Table 1:** Comparison of exercise-induced changes in pulmonary versus systemic vascular resistance and pressure. The pulmonary vasculature has very low resistance at rest but relatively limited capacity for further reduction. Therefore, exercise-induced increases in flow result in greater pressures due to the inability to compensate by reducing resistance. As described by the simplified Poiseuille's law: Pressure = Flow × Resistance.

	Left Ventricle	Right Ventricle
Rest		

Cardiac Output (L/min)	5	5
Vascular resistance (dyne-sec·cm^5^)	1100	70
Afterload Pressure (mmHg)	130/75 (85)	25/9 (15)

Exercise		

Cardiac Output (L/min)	25	25
Vascular resistance (dyne-sec·cm^5^)	↓↓↓	↓
Afterload Pressure (mmHg)	↑	↑↑↑
